# Routinely assessing patients' sleep health is time well spent

**DOI:** 10.1016/j.pmedr.2019.100851

**Published:** 2019-03-15

**Authors:** Jean-Philippe Chaput, Judy Shiau

**Affiliations:** a Healthy Active Living and Obesity Research Group, CHEO Research Institute, Ottawa, Ontario, Canada; b Department of Pediatrics, Faculty of Medicine, University of Ottawa, Ottawa, Ontario, Canada; c LEAF Weight Management Clinic, Ottawa, Ontario, Canada; d Division of Endocrinology and Metabolism, Department of Medicine, Faculty of Medicine, University of Ottawa, Ottawa, Ontario, Canada

**Keywords:** Sleep, Lifestyle, Clinic, Assessment, Public health

## Abstract

Poor sleep health is common in today's society and contributes to a wide array of health problems, decreases productivity, and increases the risk of accidents. Key sleep characteristics that should be assessed by clinicians include *sleep duration*, *sleep quality*, *sleep timing*, *daytime alertness*, and the *absence of a sleep disorder*. Examples of questions to be used by busy clinicians to quickly assess a patient's sleep health are provided. It is hoped that sleep health will be given the same level of attention as diet and exercise in clinic.

## Highlights

• Poor sleep health is pervasive in today's society.
• Poor sleep health is an important roadblock in lifestyle and health-promoting interventions.
• Simple questions can be used in clinic to assess a patient's sleep health.
• Clinicians should discuss sleep health and initiate treatment options with their patients.

Poor sleep health has become a common concern in our overworked, over-stimulated society. In fact, a third of adults in the United States (Liu et al., 2016) and Canada (Chaput et al., 2017a) sleep less than the 7 h per night, as recommended by public health authorities for optimal health benefits. Unfortunately, inadequate sleep is associated with a wide range of adverse health problems including obesity, type 2 diabetes, cardiovascular disease and depression. Further, inadequate sleep can lead to mistakes in the workplace, lower psychomotor performance, and increased risk for motor vehicle crashes. All together, poor sleep health is a significant burden to our health and economic sectors in injury and disability each year (Institute of Medicine (US) Committee on Sleep Medicine and Research et al., 2006).

The concept of “sleep health” is gaining momentum globally, and posits sleep characteristics on a continuum and not only as the presence or absence of sleep disorders (Buysse, 2014). The integration and prioritization of sleep health into the public health arena is under way (Chaput, in press), with a goal that it will become an equal counterpart to the attention and resources given to other lifestyle behaviors such as nutrition and physical activity. However, sleep health is rarely assessed in clinic. Yet, poor sleep health can be an important roadblock in many lifestyle and health-promoting interventions, and sleep health should be included as part of the lifestyle and general health assessment to the same level of attention as diet and exercise (St-Onge et al., 2016; Grandner and Malhotra, 2015). Sleep health can affect nutrition and physical activity, and this should also be assessed in clinic. This strategy aligns with an integrated approach to health (i.e., the whole day matters) that aims to examine the interconnections among all lifestyle behaviors and their combined influence on health outcomes (Chaput et al., 2017b).

Although a clinician may obtain a snapshot of whether sleep is an issue by asking “How is your sleep in general?”, this simple question risks underdiagnosing unrecognized sleep patterns that are negatively impacting a patient's health. Ideally, five key sleep characteristics should be assessed: *sleep duration*, *sleep quality*, *sleep timing*, *daytime alertness*, and the *absence of a sleep disorder*. Examples of questions to be used in clinic are provided (see Table 1). These questions have been selected from previous sleep health questionnaires, including the National Sleep Foundation's Sleep Health Index and the National Healthy Sleep Awareness Project. They are by no means comprehensive to fully assess the complexity of sleep but are meant to be practical for busy clinicians to quickly assess a patient's sleep health.

**Table 1:** Six simple questions that can be included in the assessment of sleep health in clinic.

Questions	Desired answers
1. How many hours do you sleep on an average night?	7–9 h per night for adults
2. During the past month, how would you rate your sleep quality overall?	Very good or fairly good
3. Do you go to bed and wake up at the same time every day, even on weekends?	Yes, consistent sleep schedule in general
4. How likely is that you would fall asleep during the daytime without intending to or that you would struggle to stay awake while you were doing things?	Unlikely
5. How often do you have trouble going to sleep or staying asleep?	Never, rarely or sometimes
6. During the past 2 weeks, for about how many days did you have loud snoring?	Never

Adapted from recent sleep health questionnaires for adults in the US (National Sleep Foundation's Sleep Health Index and National Healthy Sleep Awareness Project). The questions aim to provide a snapshot of sleep health in <2 min and give key information about five sleep health dimensions: sleep duration (question 1), sleep quality (question 2), sleep timing (question 3), daytime alertness (question 4), and a possible sleep disorder such as insomnia (question 5) or obstructive sleep apnea (question 6). Question 1 can be adapted for shift workers (because they sleep during the day) and for those taking naps (in which case nap duration can be added to the nightly sleep duration). Other aspects of sleep health can be considered if time permits. For example, one may want to question about social jet lag, or the discrepancy between sleep on work and free days, or weekdays vs. weekends. A high discrepancy is not desired (biological and social times should be aligned as much as possible and work and activity schedules should be adapted to the chronotype of each individual whenever possible). Red flag answers from the sleep assessment above include things such as “I snore so loud my partner sleeps in another room”, “I wake up gasping and choking”, “I fall asleep when driving”, “I sleep walk”, “I am awake between 2:00 am and 4:00 am”, “I wake up and eat at night”, “I drink alcohol or take Gravol to fall asleep” etc.

Concerning answers should illicit a thorough history to identify root causes and barriers of healthy sleep. Factors could include excessive screen time, blue light exposure, consumption of wake-promoting substances, long work hours, shift work, family demands, social activities, and intercontinental travel. Screening for common sleep disorders should also be part of this sleep health assessment, because poor sleep health can be a consequence of sleep disorders. Among the solutions to improve sleep health, multicomponent behavioral interventions have shown to provide good results (Van Dyk et al., in press), especially individualized, pragmatic, problem-solving approaches using established behavioral principles as well as evidence-based treatment of sleep disorders.

Most patients will provide at least one undesired answer to the questions in Table 1. There is also a gradation in the severity of sleep health problems, with more attention and intense treatment needed with greater sleep problems (e.g., snoring once a week vs. every night). Whether clinicians should address the sleep issue first with behavioral interventions or provide a referral to a sleep specialist depends on: (i) What the sleep issue is and what are the contributing factors; (ii) Whether there is a suspected sleep disorder; and (iii) Whether the clinician has the training/competence to address the sleep issue. The informed decision-making approach for the order and nature of actions should be based on the information obtained during history taking. If the main contributing factors to poor sleep health appear to be behavioral/lifestyle/environmental, it could be addressed using targeted recommendations/interventions (ideally accompanied by qualified health care providers and a multidisciplinary team to evaluate and monitor the evolution of the patient). If there is indication that there may be an underlying sleep disorder, the patient should be provided evidence-based assessment and interventions according to clinical practice guidelines (and this may require a referral to a sleep specialist).

Suggestions on what to do for the questions listed in Table 1 include:

Q1, Q2, Q3 and Q4: Ascertain why the patient is not getting enough sleep and of sufficient quality. Ascertain why the patient does not have a consistent or regular bedtime and wake-up routine. Address sleep hygiene (e.g., removing screens from the bedroom, reducing screen time exposure during the day, increasing physical activity level, making sure the bedroom is dark, quiet, comfortable and cool, reducing caffeine consumption, having a relaxing bedtime routine) if sleep deprivation or poor sleep quality is caused by poor sleep habits or modifiable environmental factors. Sleep hygiene should be individually tailored; for example, if a patient is sleep deprived because of non-modifiable environmental factors or life circumstances, naps can be helpful and sometimes essential for safety and global health. If the patient screens positive for common sleep disorders (e.g., insomnia or obstructive sleep apnea [OSA]), these sleep disorders should be assessed and treated (sleep hygiene is not an evidence-based treatment for sleep disorders).

Q5 (insomnia): If the patient has insomnia disorder the first-line treatment is cognitive behavioral therapy (CBT) (van Straten et al., 2018). Pharmacological options can be considered if CBT for insomnia has not solved the problem or if the patient does not want to or cannot pursue CBT.

Q6 (possible OSA): Red flags include loud snoring, episodes of breathing cessation during sleep, abrupt awakenings accompanied by gasping or choking, morning headache, and enlarged neck circumference (men: >43 cm [17 in.] and women: >37 cm [15 in.]). Order a sleep study if an OSA is suspected. If positive, referrals can be sent to a sleep physician who will consider evidence-based treatment options (e.g., continuous positive airway pressure [CPAP] machine) or the clinician can refer the patient to a dentist if oral appliance is a possible treatment option based on the characteristics of OSA and patient preference.

Just like any behavior change, it is important to identify the drivers for motivating the patient to make change. Is it to decrease fatigue? Is it to improve daytime functioning? Is it to help with weight? For example, individuals not getting enough sleep eat more, engage in more screen time, and tend to move less (Chaput, 2014). As a result, sleep deprivation has been shown to cause weight gain and obesity-related problems (Chaput, 2014). Although maintaining good sleeping habits is increasingly recognized as a good strategy to prevent future weight gain, healthy sleep is also important to improve the success of weight-loss interventions (Chaput and Tremblay, 2012). This means that patients not getting sufficient sleep can expect to lose less body fat compared to those who sleep the recommended amount for the same weight-loss program (Wang et al., in press). The idea that inadequate sleep may undermine the efficacy of weight-loss interventions is increasingly recognized and accepted by health care professionals and has found its way into clinical practice. For example, Obesity Canada (the largest obesity organization in the world) has endorsed the concept of addressing sleep for successful weight management in their set of practitioner tools – the 5As of Obesity Management (ask, assess, advise, agree and assist) (https://obesitycanada.ca/resources/5as/).

In summary, despite the well-known consequences of inadequate sleep on health, safety, and performance, sleep is often perceived as a luxury or a waste of time. There is strong evidence demonstrating the benefits of good sleep health and clinicians should be encouraged to discuss sleep and initiate treatment options with their patients. Unfortunately, sleep difficulties in primary care are often mismanaged and often not investigated at all (Ulmer et al., 2017). Addressing poor sleep health has proven to result in beneficial effects on quality of life and many health outcomes through positive impacts on physiological mechanisms and other lifestyle behaviors such as diet and physical activity.
